# Association of oxidative balance score and lung health from the National Health and Nutrition Examination Survey 2007–2012

**DOI:** 10.3389/fnut.2022.961950

**Published:** 2023-01-09

**Authors:** Zhixiao Xu, Yincong Xue, Hezhi Wen, Chengshui Chen

**Affiliations:** ^1^Department of Pulmonary and Critical Care Medicine, The First Affiliated Hospital of Wenzhou Medical University, Wenzhou, China; ^2^Key Laboratory of Interventional Pulmonology of Zhejiang Province, Wenzhou, China; ^3^The Quzhou Affiliated Hospital of Wenzhou Medical University, Quzhou People’s Hospital, Quzhou, China

**Keywords:** oxidative balance score, oxidative stress, lung health, spirometry, diet, lifestyle

## Abstract

**Background:**

Oxidative stress is associated with outcomes of chronic lung disease. The oxidative stress-related exposures of diet and lifestyle can be evaluated by the oxidative balance score (OBS), and higher OBS scores indicate more significant antioxidant exposures. But the relationship between OBS and lung health is unknown.

**Purpose:**

The aim of this study was to explore the association between OBS and lung health (respiratory symptoms, chronic lung disease, and lung function).

**Methods:**

A series of models, including weighted linear models, weighted logistic regression, and weighted multinomial logistic regression, were performed to assess the associations of OBS with respiratory symptoms, chronic lung disease, and lung function. The models adjusted by age, race/ethnicity, gender, educational background, poverty-to-income ratio, and dietary energy were also performed.

**Results:**

Cross-sectional data of 5,214 participants from the National Health and Nutrition Examination Survey for the years 2007–2012 were analyzed. For every one-unit increase in OBS, the odds of wheezing/chronic bronchitis decreased by 6%. Increased OBS was associated with higher percent-predicted forced expiratory volume in one second (FEV1) (adjusted mean difference (MD), 0.21%; 95% CI: 0.10–0.32) and percent-predicted forced vital capacity (FVC) (adjusted MD, 0.15%; 95% CI: 0.07–0.24). A significantly lower risk of wheezing/chronic bronchitis was found in participants in the second/third/fourth OBS quartile compared to those in the first OBS quartile (all *P* for trend < 0.05). Moreover, higher percent-predicted FEV1 and FVC were also found in the third quartile and fourth quartile (all *P* for trend < 0.05). Furthermore, both dietary and lifestyle components were tightly related to pulmonary outcomes. Many associations were maintained after stratified by sex or after sensitivity analyses.

**Conclusion:**

Oxidative balance score was negatively correlated with the diagnosis of chronic bronchitis/wheezing/restrictive spirometry pattern and positively correlated with percent-predicted FVC and FEV1. It seems that the higher the OBS score, the better the pulmonary outcomes. The findings highlight the importance of adherence to an antioxidant diet and lifestyle and that it contributes to lung health.

## 1. Introduction

Oxidative stress is regarded as an imbalance between oxidant and antioxidant effects in the body and has been linked to a wide range of diseases such as impaired pulmonary function (1, 2), asthma, and chronic pulmonary obstructive disease (COPD) (3–8). Oxidative stress plays an important role in the pathophysiology of respiratory diseases, especially due to the direct exposure of respiratory tissues to oxidants in ambient air (9). For example, exogenous factors such as environmental pollutants and cigarette smoke could lead to elevated levels of oxidative stress in patients (10, 11). While oxidative stress is probably a contributing factor to impaired lung function, the body’s ability to resist oxidative stress should also be paid attention to. There was evidence that dietary antioxidants, especially vitamin C and β-carotene, were also associated with smoking (12). Moreover, associations between the intake of antioxidant vitamins (e.g., vitamin C, vitamin E, and β-carotene) or fibers and respiratory diseases have been demonstrated (2, 13–16), and various antioxidants (vitamins and β-carotene) were positively correlated with forced expiratory volume in one second (FEV1) and forced vital capacity (FVC) (1, 9), but some pro-oxidants (thiobarbituric acid-reactive substance) were linked with lower lung function (17, 18). Therefore, exposure to antioxidant nutrients that could inhibit oxidizing radicals should be taken into important part (9).

The oxidative balance score (OBS), developed as a composite measure of individual oxidative homeostasis, was determined by a combination of pro-oxidant and antioxidant factors (19). OBS, which commonly involved dietary components and lifestyle-related components, was extensively used in epidemiological studies to evaluate the interaction between oxidative status and the risk of development of multiple chronic diseases (20). Previous studies have demonstrated that higher OBS was related to better glycemic control in Iranian adults with type 2 diabetes (21). A positive association was observed between the total OBS and quality of life in patients with osteoarthritis (22). In the Chronic Renal Insufficiency Cohort, oxidative balance-related exposures were not related to chronic kidney disease progression or cardiovascular disease risk (23). Therefore, the relationship between OBS and chronic diseases, especially respiratory diseases, still needs to be further explored.

Although both physicians and patients are keen to investigate the potential effects of antioxidant nutrients and lifestyle on respiratory diseases, the association between OBS and lung health has not yet been evaluated. Identifying the association between OBS and lung health would pave the way for future patient education and therapeutic strategies. This study aimed to investigate the relationship between OBS and lung health.

## 2. Materials and methods

### 2.1. Study population

**FIGURE 1:**
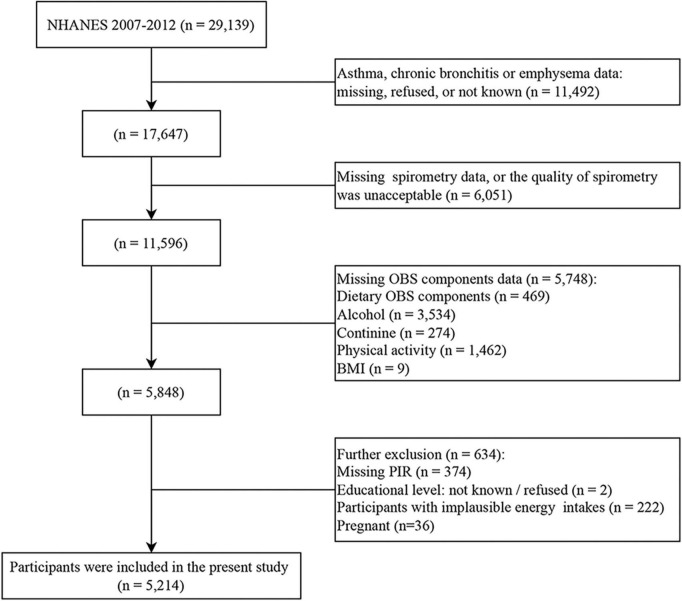
Flowchart depicting the cohort participant selection.

The present study was an observational population-based study using data from the National Health and Nutrition Examination Survey (NHANES). NHANES is a national survey of children and adults in the United States conducted every 2 years, but only three 2-year survey cycles (2007–2008, 2009–2010, and 2011–2012) include information on spirometry. Among 29,139 participants in the NHANES 2007–2012, individuals were excluded if (1) they lost data for any of the conditions, including asthma, chronic bronchitis, and emphysema (*n* = 11,492), (2) they lost data for any of spirometry, or the quality of spirometry was unacceptable (*n* = 6,051), (3) they lost data for any of the OBS components (*n* = 5,748), and (4) further exclusion included improper energy intake, pregnant and several missing covariates data (*n* = 634). In the study, improper energy intake was defined as <800 kcal/d or > 4200 kcal/d for men and <500 kcal/d or > 3500 kcal/d for women (24). Finally, a total of 5,214 participants were recruited for the present study (Figure 1).

National Health and Nutrition Examination Survey was conducted with approval by the National Center for Health Statistics Ethics Review Board, and all participants gave written informed consent.

### 2.2. Exposure definitions

Based on *a priori* information (24), a range of components have been used to calculate OBS, and 16 nutrients and four lifestyle factors were screened in the present study. The overall OBS consisting of five pro-oxidants and 15 antioxidants was calculated by adding the scores attributed to each component. Higher OBS scores indicated more significant antioxidant exposures.

NHANES participants who completed at least one valid dietary recall were recruited. Dietary intake data were derived from an average of two 24-h dietary interviews (the first dietary recall interview was conducted in person, and the second interview was collected by telephone 3–10 days later). The types and amounts of food and beverages, as well as energy, nutrients, and other food components (excluding dietary supplements or medications), were estimated. In this study, lifestyle factors were incorporated into physical activity, alcohol consumption, body mass index (BMI), and smoking. Physical activity was represented by metabolic equivalent (MET) scores × weekly frequency of each physical activity × duration of each physical activity (25). The physical activities included work-related activity (vigorous intensity work-related activity and moderate intensity work-related activity) and leisure-time physical activity (walking or bicycling for transportation, vigorous leisure-time physical activity, and moderate leisure-time physical activity). Data on alcohol consumption were obtained from the question “In the past 12 months, on those days that you drank alcoholic beverages, on average, how many drinks did you have?”. Body measurements were recorded for all participants by trained examiners. To give equal attention to active and passive smoking, serum cotinine which was the major metabolite of nicotine was used to reflect smoking. The processes of cotinine processing, storage, and analysis were described in detail on the NHANES website, and isotope-dilution high-performance liquid chromatography/atmospheric pressure chemical ionization tandem mass spectrometry (ID HPLC-APCI MS/MS) was used to measure serum cotinine.

All components were divided into three groups by weighted tertiles. For the groups from the first tertile to the third tertile, antioxidants were assigned fractions from 0 to 2, respectively, while pro-oxidants were assigned fractions from 2 to 0 (Table 1).

**TABLE 1:** Components of the oxidative balance score.

OBS components	Property	Male			Female		
		0	1	2	0	1	2
**Dietary OBS components**							
Dietary fiber (g/d)	A	<13.95	13.4–21.75	≥21.75	<12.00	12.00–18.45	≥18.45
Carotene (RE/d)	A	<631	631–2,051.5	≥2,051.5	<679.5	679.5–2,360.5	≥2,360.5
Riboflavin (mg/d)	A	<1.92	1.92–2.73	≥2.73	<1.50	1.50–2.13	≥2.13
Niacin (mg/d)	A	<24.83	24.83–34.32	≥34.32	<17.28	17.28–23.97	≥23.97
Vitamin B6 (mg/d)	A	<1.89	1.89–2.74	≥2.74	<1.36	1.36–2.00	≥2.00
Total folate (mcg/d)	A	<356	356–517.5	≥517.5	<287	287–407	≥407
Vitamin B12 (mcg/d)	A	<4.11	4.11–6.83	≥6.83	<2.80	2.80–4.82	≥4.82
Vitamin C (mg/d)	A	<42.75	42.75–103.75	≥103.75	<40.10	40.10–91.00	≥91.00
Vitamin E (ATE) (mg/d)	A	<6.33	6.33–10.07	≥10.07	<5.31	5.31–8.20	≥8.20
Calcium (mg/d)	A	<809	809–1,229	≥1,229	<675.5	675.5–1,012.5	≥1,012.5
Magnesium (mg/d)	A	<282.5	282.5–382	≥382	<225.5	225.5–313.5	≥313.5
Zinc (mg/d)	A	<10.70	10.70–15.44	≥15.44	<7.73	7.73–10.79	≥10.79
Copper (mg/d)	A	<1.14	1.14–1.62	≥1.62	<0.96	0.96–1.32	≥1.32
Selenium (mcg/d)	A	<108.65	108.65–148.20	≥148.20	<75.90	75.90–104.85	≥104.85
Total fat (g/d)	P	≥104.64	73.42–104.64	<73.42	≥76.13	53.47–76.13	<53.47
Iron (mg/d)	P	≥18.77	13.44–18.77	<13.44	≥14.49	10.39–14.49	<10.39
**Lifestyle OBS components**							
Physical activity (MET–minute/week)	A	<1,800	1,800–5,280	≥5,280	<960	960–3,020	≥3,020
Alcoholic drinks at past 12 months (drink/d)	P	>3	2–3	≤2	>2	1–2	≤1
Body mass index (kg/m2)	P	≥29.90	25.72–29.90	<25.72	≥28.97	23.96–28.97	<23.96
Cotinine (ng/mL)	P	≥1.950	0.024–1.950	<0.024	≥0.015	0.015–0.131	<0.131

Antioxidant was represented by A; pro-oxidant was represented by P; retinol equivalent was represented by RE; alpha-tocopherol equivalent was represented by ATE; metabolic equivalent represented by MET.

### 2.3. Outcome definitions

Respiratory symptoms, chronic lung disease, and lung function were enrolled as outcomes in this study, where respiratory symptoms included cough, sputum production, wheezing, and exertional dyspnea while chronic lung disease was comprised of asthma, bronchitis, and emphysema.

The presence of cough, phlegm production, wheezing, and exertional dyspnea was determined by the responses to the following questions, respectively. The questions included, “Do you usually cough on most days for 3 consecutive months or more during the year?”, “Do you bring up phlegm on most days for 3 consecutive months or more during the year?”, “In the past 12 months have you had wheezing or whistling in your chest?”, and “Have you had shortness of breath either when hurrying on the level or walking up a slight hill?”.

Regarding the presence of chronic lung disease, the question “Has a doctor or other health professional ever told you that you had asthma?” was used to determine the presence or absence of asthma. The question “Ever told you had chronic bronchitis?” was used to determine the presence or absence of chronic bronchitis. Similarly, the question “Ever told you had emphysema?” was also used for emphysema.

### 2.4. Lung function measures

From NHANES 2007 to 2012, participants who met inclusion criteria were invited to undergo prebronchodilator spirometry, which was performed using the Ohio 822/827 dry rolling volume sealed spirometer. The FEV1 and FVC were measured, and only results under American Thoracic Society (ATS) quality class B or higher (meets ATS data collection standards) were recruited. Predicted values were derived based on the Hankinson equation (26). The obstructive spirometry model was determined as FEV1/FVC < 0.70, and the restrictive spirometry model was defined as FEV1/FVC ≥ 0.70 and predicted FVC < 80%.

### 2.5. Covariate definitions

The covariates included age, race/ethnicity, gender, educational background, and poverty-to-income ratio (PIR). The race/ethnicity was categorized as non-Hispanic white, non-Hispanic black, Mexican American, and others. Education background was graded into less than 9th grade, 9–11th grade (including 12th grade with no diploma), high school grade/general equivalent diploma, some college or associate degree, and college graduate or above.

### 2.6. Data analysis

In the process of baseline characterization, the continuous variables were tested for normal distribution, and all were non-normal continuous variables. Therefore, median (IQR) and unweighted frequencies (weighted percentages) were used to represent non-normal continuous and categorical variables, respectively. To test for differences in the characteristics of the variables across different OBS groups (quartiles), the Rao–Scott chi-squared test and the Kruskal–Wallis test were used for categorical and non-normal continuous variables, respectively.

Weighted linear models and weighted logistic regression were used to assess associations of OBS with dichotomous and continuous outcomes, respectively. Weighted multinomial logistic regression was conducted to assess the relative risk ratio between OBS and an obstructive or restrictive spirometry pattern, with a normal pattern as the referent group. In addition, we considered diet weight (provided by NHANES) in our analyses. Model 1 was the crude model without adjustment for any potential confounders. Model 2 adjusted for age, sex, race/ethnicity, education level, PIR, and dietary energy. All regressions accounted for survey weights.

Oxidative balance score has been divided into diet OBS and lifestyle OBS to explore their association with lung health, respectively. Given the established gender differences in diet, the analyses were repeated and stratified by sex. Moreover, to test the robustness of our findings, a sensitivity analysis was performed by replacing the dietary weight with the interview weight (27).

A two-sided *P*-value of <0.05 was defined as statistically significant. Analyses were performed in R 4.1.1.

## 3. Results

### 3.1. Baseline characteristics

The baseline characteristics of individuals by quartiles of the OBS are shown in Table 2, with the majority of participants being non-Hispanic white. Although there were several missing data on respiratory symptoms, there was a high prevalence of cough (8.3%), sputum production (6.5%), wheezing (12%), and exertional dyspnea (43%), similar to those reported in previous studies (28). Notably, the prevalence of airway obstruction detected by spirometry (12%) was higher than the prevalence of self-reported chronic bronchitis (4.0%) and emphysema (0.5%). But a small number of individuals revealed a restrictive pattern in spirometry (4.1%).

**TABLE 2:** Baseline characteristics of participants by quartiles of the oxidative balance score.

	ALL	Q1 (<15)	Q2 (15–20)	Q3 (20–26)	Q4 (≥26)	*P*-value
	***N* = 5,214**	***N* = 1,470**	***N* = 1,162**	***N* = 1,368**	***N* = 1,214**	
Age	42 [30;54]	40 [28;52]	43 [30;53]	44 [30;55]	44 [32;56]	<0.001
Gender						0.114
Male	2,876 (53%)	797 (51%)	659 (54%)	779 (56%)	641 (50%)	
Female	2,338 (47%)	673 (49%)	503 (46%)	589 (44%)	573 (50%)	
Race/ethnicity						<0.001
Mexican American	736 (6.9%)	213 (8.0%)	149 (6.8%)	227 (7.6%)	147 (5.4%)	
Other Hispanic	483 (4.6%)	144 (5.2%)	100 (4.6%)	132 (4.7%)	107 (3.9%)	
Non-Hispanic White	2,699 (75%)	649 (67%)	594 (74%)	714 (77%)	742 (82%)	
Non-Hispanic Black	887 (8.1%)	374 (15%)	217 (8.5%)	182 (6.3%)	114 (3.7%)	
Other race - including multi-racial	409 (5.1%)	90 (5.1%)	102 (6.2%)	113 (4.5%)	104 (4.9%)	
Education level						<0.001
Less than 9th grade	287 (2.4%)	111 (3.6%)	53 (2.3%)	80 (2.6%)	43 (1.0%)	
9–11th grade (includes 12th grade with no diploma)	611 (8.2%)	240 (12%)	156 (10%)	138 (7.3%)	77 (3.7%)	
High school grad/GED or equivalent	1,084 (19%)	385 (27%)	258 (20%)	259 (18%)	182 (12%)	
Some college or AA degree	1,658 (33%)	487 (37%)	382 (36%)	420 (30%)	369 (32%)	
College graduate or above	1,574 (37%)	247 (21%)	313 (31%)	471 (42%)	543 (51%)	
Ratio of family income to poverty	3.69 [1.81;5.00]	2.58 [1.24;4.63]	3.51 [1.80;5.00]	4.10 [2.06;5.00]	4.59 [2.40;5.00]	<0.001
Energy (kcal)	2,057 [1,570;2,699]	1,626 [1,222;2.56]	1,936 [1,432;2,458]	2,249 [1,774;2,832]	2,499 [1,969;3,149]	<0.001
Asthma						0.867
No	4,459 (85%)	1,232 (85%)	1,003 (86%)	1,166 (85%)	1,058 (86%)	
Yes	755 (15%)	238 (15%)	159 (14%)	202 (15%)	156 (14%)	
Chronic bronchitis						0.003
No	5,002 (96%)	1,381 (94%)	1,123 (97%)	1,317 (96%)	1,181 (98%)	
Yes	212 (4.0%)	89 (6.4%)	39 (3.4%)	51 (4.1%)	33 (2.4%)	
Emphysema						0.045
No	5,177 (99%)	1,452 (99%)	1,153 (100%)	1,361 (100%)	1,211 (100%)	
Yes	37 (0.5%)	18 (1.0%)	9 (0.5%)	7 (0.5%)	3 (0.2%)	
FVC (mL)	4,240 [3,563;5,067]	4,060 [3,471;4,872]	4,175 [3,531;5,052]	4,307 [3,623;5,108]	4,371 [3,625;5,198]	<0.001
FEV1 (mL)	3,352 [2,795;3,991]	3,216 [2,722;3,847]	3,364 [2,775;3,979]	3,392 [2,854;4,009]	3,413 [2,839;4,086]	<0.001
FEV1/FVC (%)	79.1 [74.3;83.4]	79.9 [74.9;84.1]	79.1 [74.4;83.3]	79.1 [74.1;82.9]	78.7 [73.6;83.0]	0.173
Percent-predicted FVC	99.8 [91.8;108.0]	98.9 [91.1;107.1]	99.3 [90.6;107.9]	100.1 [92.2;108.1]	101.1 [93.5;108.8]	<0.001
Percent-predicted FEV1	97.6 [89.5;106.1]	96.1 [89.1;104.4]	97.3 [88.2;106.5]	98.1 [89.7;106.7]	98.3 [91.0;106.8]	<0.001
Spirometry pattern						0.070
No	4,315 (84%)	1,186 (82%)	966 (83%)	1,137 (84%)	1,026 (85%)	
Obstructive	622 (12%)	178 (12%)	131 (11%)	168 (13%)	145 (12%)	
Restrictive	277 (4.1%)	106 (5.6%)	65 (5.3%)	63 (3.2%)	43 (2.6%)	
Coughing most days in over 3 months						0.109
No	2,611 (92%)	660 (91%)	582 (89%)	714 (92%)	655 (94%)	
Yes	245 (8.3%)	82 (8.7%)	59 (11%)	59 (8.2%)	45 (6.4%)	
Bring up phlegm most days in 3 months						0.043
No	2,652 (94%)	676 (93%)	590 (91%)	731 (95%)	655 (95%)	
Yes	204 (6.5%)	66 (7.2%)	51 (8.8%)	42 (5.3%)	45 (5.3%)	
Wheezing or whistling in chest in the past 12 months						<0.001
No/Not known	4,562 (88%)	1,224 (84%)	1,016 (87%)	1,213 (90%)	1,109 (92%)	
Yes	652 (12%)	246 (16%)	146 (13%)	155 (10%)	105 (7.9%)	
Chest sound wheezy during exercise						0.184
No/Not known	353 (57%)	108 (48%)	87 (60%)	91 (60%)	67 (63%)	
Yes	299 (43%)	138 (52%)	59 (40%)	64 (40%)	38 (37%)	

FVC, forced vital capacity; FEV1, forced expiratory volume in one second.

Participants in the highest OBS quartile tended to have higher educations, higher PIR, and higher total energy intakes compared to those in the lowest OBS quartile. Both FVC and FEV1 were higher in the fourth OBS quartile when compared with the first OBS quartile as well as percent-predicted FEV1 and FVC had significant trends from the low OBS quartile to the high OBS quartile. Moreover, there were significant differences in the prevalence of self-reported chronic bronchitis and emphysema, as well as the prevalence of phlegm production and wheezing for OBS quartiles. But there was no significant trend in the prevalence of self-reported asthma from the low OBS quartile to the high OBS quartile. The prevalence of cough was presented similarly (Table 2).

### 3.2. Association between the OBS and study outcomes

With respect to self-reported chronic lung disease, the population for the diagnosis of emphysema was too small, so the logistic regression analysis was not performed to avoid errors. However, higher OBS was not significantly related to the odds of a diagnosis of asthma but was significantly related to the odds of a diagnosis of chronic bronchitis (adjusted odds ratio (OR), 0.94; 95% CI: 0.91–0.97). Regarding self-reported respiratory symptoms, an association between OBS with wheezing was revealed even in adjusted analysis (Table 3). For every one-unit increase in OBS, the odds of wheezing decreased by 6%. However, the association between OBS and the diagnosis of cough was only present in the adjusted model, and the association between OBS and the diagnosis of exertional dyspnea was only present in the unadjusted model (Table 3), which might be due to some confounding factors.

**TABLE 3:** Association of oxidative balance score with study outcomes.

	Unadjusted	*P*-value	Adjusted	*P*-value
**Condition, OR (95% CI)**				
Asthma	0.995 (0.98 to 1.01)	0.603	0.99 (0.97 to 1.01)	0.443
Chronic bronchitis	0.95 (0.93 to 0.97)	<0.001	0.94 (0.91 to 0.97)	<0.001
**Symptom, OR (95% CI)**				
Cough	0.98 (0.95 to 1.002)	0.065	0.95 (0.92 to 0.97)	<0.001
Phlegm production	0.97 (0.95 to 1.00)	0.054	0.96 (0.91 to 1.01)	0.123
Wheeze	0.96 (0.94 to 0.97)	<0.001	0.94 (0.92 to 0.96)	<0.001
Exertional dyspnea	0.96 (0.93 to 0.997)	0.036	0.97 (0.93 to 1.01)	0.172
**Spirometry, MD (95% CI)**				
FEV1/FVC	−0.04 (−0.08 to 0.01)	0.103	0.05 (0.005 to 0.09)	0.031
Percent-predicted FVC	0.13 (0.07 to 0.19)	<0.001	0.15 (0.07 to 0.24)	<0.001
Percent-predicted FEV1	0.15 (0.08 to 0.23)	<0.001	0.21 (0.10 to 0.32)	<0.001
**Spirometry pattern, RRR (95% CI)**				
Obstructive	1.00 (0.98 to 1.02)	0.971	0.98 (0.96 to 1.01)	0.148
Restrictive	0.96 (0.94 to 0.99)	0.002	0.95 (0.92 to 0.98)	0.003

OR, odds ratio; MD, mean difference; RRR, relative risk ratio; CI, confidence intervals; FVC, forced vital capacity; FEV1, forced expiratory volume in one second.

The adjusted models were adjusted by age, sex, race/ethnicity, poverty-to-income ratio, and dietary energy.

For prebronchodilator spirometry, increased OBS was associated with higher percent-predicted FEV1 (adjusted mean difference (MD), 0.21%; 95% CI: 0.10–0.32) and percent-predicted FVC (adjusted MD, 0.15%; 95% CI: 0.07–0.24). Increased OBS was associated with higher FEV1/FVC (adjusted MD, 0.05%; 95% CI: 0.005–0.09), even if the association was subtle. Furthermore, higher OBS was correlated with a lower relative risk of a restrictive spirometry pattern (relative risk ratio (RRR), 0.96; 95% CI: 0.94–0.99), even after adjustment (adjusted RRR, 0.95; 95% CI: 0.92–0.98) (Table 3).

### 3.3. Independent effect of OBS quartiles on study outcomes

An SD increase in OBS was associated with a decrease in the adjusted risk of the diagnosis of chronic bronchitis (adjusted OR: 0.63, 95% CI: 0.50–0.81) (Table 4). Consistently, when OBS was assessed as quartiles, compared with the first quartile OBS, the adjusted ORs for chronic bronchitis of the second quartile OBS, third quartile OBS and fourth quartile OBS were 0.47 (0.27–0.82), 0.58 (0.35–0.99), and 0.28 (0.12–0.64), respectively; importantly, *P* for trend < 0.05 indicated a linear trend in the quartiles of OBS with the diagnosis of chronic bronchitis. Moreover, the association between the diagnosis of wheezing and OBS was presented similarly (Table 4). Furthermore, there was a statistically significant association between the diagnosis of cough and the fourth quartile OBS.

**TABLE 4:** Association of quartiles of oxidative balance score on study outcomes.

	Unadjusted	*P*-value	Adjusted	*P*-value
**Condition, OR (95% CI)**				
**Asthma**				
OBS per SD	0.97 (0.85 to 1.10)	0.603	0.94 (0.80 to 1.11)	0.443
Q1	Ref	Ref	Ref	Ref
Q2	0.92 (0.69 to 1.23)	0.572	0.92 (0.66 to 1.28)	0.601
Q3	0.94 (0.70 to 1.27)	0.695	0.92 (0.66 to 1.30)	0.639
Q4	0.88 (0.63 to 1.24)	0.456	0.81 (0.53 to 1.23)	0.305
*P* for trend		0.486		0.311
**Chronic bronchitis**				
OBS per SD	0.71 (0.60 to 0.83)	<0.001	0.63 (0.50 to 0.81)	<0.001
Q1	Ref	Ref	Ref	Ref
Q2	0.51 (0.31 to 0.84)	0.010	0.47 (0.27 to 0.82)	0.009
Q3	0.63 (0.39 to 1.02)	0.061	0.58 (0.35 to 0.99)	0.045
Q4	0.36 (0.20 to 0.63)	<0.001	0.28 (0.12 to 0.64)	0.004
*P* for trend		<0.001		0.003
**Symptom, OR (95% CI)**				
**Cough**				
OBS per SD	0.84 (0.70 to 1.01)	0.065	0.67 (0.54 to 0.82)	<0.001
Q1	Ref	Ref	Ref	Ref
Q2	1.27 (0.81 to 1.99)	0.294	1.04 (0.66 to 1.62)	0.871
Q3	0.94 (0.60 to 1.49)	0.788	0.69 (0.45 to 1.05)	0.081
Q4	0.72 (0.41 to 1.29)	0.266	0.43 (0.24 to 0.79)	0.008
*P* for trend		0.126		0.001
**Phlegm production**				
OBS per SD	0.82 (0.68 to 1.003)	0.054	0.74 (0.51 to 1.09)	0.123
Q1	Ref	Ref	Ref	Ref
Q2	1.24 (0.78 to 1.99)	0.358	1.20 (0.72 to 2.00)	0.481
Q3	0.72 (0.42 to 1.21)	0.204	0.66 (0.31 to 1.38)	0.260
Q4	0.71 (0.42 to 1.20)	0.195	0.59 (0.24 to 1.48)	0.252
*P* for trend		0.059		0.146
**Wheeze**				
OBS per SD	0.73 (0.64 to 0.83)	<0.001	0.63 (0.54 to 0.74)	< 0.001
Q1	Ref	Ref	Ref	Ref
Q2	0.79 (0.61 to 1.02)	0.067	0.72 (0.53 to 0.98)	0.035
Q3	0.59 (0.45 to 0.78)	<0.001	0.50 (0.36 to 0.68)	<0.001
Q4	0.44 (0.31 to 0.63)	<0.001	0.32 (0.20 to 0.50)	<0.001
*P* for trend		<0.001		<0.001
**Exertional dyspnea**				
OBS per SD	0.76 (0.60 to 0.98)	0.036	0.81 (0.59 to 1.10)	0.172
Q1	Ref	Ref	Ref	Ref
Q2	0.63 (0.34 to 1.14)	0.122	0.71 (0.38 to 1.34)	0.278
Q3	0.61 (0.34 to 1.11)	0.103	0.70 (0.38 to 1.28)	0.239
Q4	0.55 (0.27 to 1.13)	0.102	0.68 (0.29 to 1.57)	0.352
*P* for trend		0.080		0.308
**Spirometry, MD (95% CI)**				
**FEV1/FVC**				
OBS per SD	−0.27 (−0.60 to 0.06)	0.103	0.36 (0.03 to 0.68)	0.031
Q1	Ref	Ref	Ref	Ref
Q2	−0.07 (−0.80 to 0.66)	0.845	0.68 (0.11 to 1.25)	0.021
Q3	−0.43 (−1.15 to 0.30)	0.245	0.76 (0.19 to 1.34)	0.011
Q4	−0.62 (−1.52 to 0.28)	0.174	0.92 (0.10 to 1.73)	0.028
*P* for trend		0.134		0.045
**Percent-predicted FVC**				
OBS per SD	0.94 (0.52 to 1.35)	<0.001	1.09 (0.48 to 1.70)	<0.001
Q1	Ref	Ref	Ref	Ref
Q2	0.41 (−0.86 to 1.67)	0.521	0.68 (−0.69 to 2.06)	0.319
Q3	1.48 (0.29 to 2.67)	0.016	1.71 (0.25 to 3.17)	0.023
Q4	2.39 (1.26 to 3.52)	<0.001	2.69 (0.97 to 4.42)	0.003
*P* for trend		<0.001		0.002
**Percent-predicted FEV1**				
OBS per SD	1.08 (0.54 to 1.63)	<0.001	1.52 (0.73 to 2.31)	<0.001
Q1	Ref	Ref	Ref	Ref
Q2	1.05 (−0.36 to 2.46)	0.140	1.56 (0.14 to 2.98)	0.032
Q3	1.97 (0.64 to 3.29)	0.004	2.62 (1.06 to 4.17)	0.002
Q4	2.84 (1.34 to 4.35)	<0.001	3.82 (1.66 to 5.98)	0.001
*P* for trend		<0.001		0.001
**Spirometry pattern, RRR (95% CI)**				
**Obstructive**				
OBS per SD	0.998 (0.88 to 1.13)	0.971	0.87 (0.73 to 1.05)	0.148
Q1	Ref	Ref	Ref	Ref
Q2	0.94 (0.69 to 1.26)	0.669	0.79 (0.58 to 1.08)	0.141
Q3	1.02 (0.75 to 1.39)	0.888	0.80 (0.56 to 1.13)	0.198
Q4	0.995 (0.70 to 1.41)	0.979	0.75 (0.48 to 1.19)	0.226
*P* for trend		0.905		0.301
**Restrictive**				
OBS per SD	0.76 (0.64 to 0.90)	0.002	0.68 (0.53 to 0.88)	0.003
Q1	Ref	Ref	Ref	Ref
Q2	0.93 (0.55 to 1.57)	0.781	0.80 (0.47 to 1.36)	0.410
Q3	0.55 (0.37 to 0.82)	0.004	0.44 (0.26 to 0.75)	0.003
Q4	0.45 (0.26 to 0.78)	0.004	0.33 (0.17 to 0.67)	0.002
*P* for trend		<0.001		<0.001

OR, odds ratio; MD, mean difference; RRR, relative risk ratio; CI, confidence intervals; FVC, forced vital capacity; FEV1, forced expiratory volume in one second.

The adjusted models were adjusted by age, sex, race/ethnicity, poverty-to-income ratio, and dietary energy.

Oxidative balance score was strongly associated with spirometry; a significantly higher percent-predicted FEV1 was found in the second, third, and fourth quartiles than participants in the first quartile (all *P* for trend < 0.05) (Table 4). There were also tight associations between percent-predicted FVC with both the third quartile OBS and the fourth quartile OBS, although after adjusted (all *P* for trend < 0.05). Although participants with the fourth OBS quartile showed a higher MD of percent-predicted FVC and FEV1 compared to those in the third OBS quartile (2.69 vs. 1.71% in percent-predicted FVC, *P* < 0.05; 3.82 vs. 2.62% in percent-predicted FEV1, *P* < 0.05), the reference group were all the first OBS quartile. There was also a statistically significant association between percent-predicted FEV1 and the second quartile OBS after adjustment. Moreover, OBS was associated with FEV1/FVC in the adjusted model.

Associations of OBS quartiles with a relative risk of an obstructive spirometry pattern were less apparent, precluding any meaningful inference. In the adjusted model, compared with the first quartile, the relative risk of the restrictive spirometry pattern was significantly decreased in the third/fourth quartile, while it was not obvious in the second quartile.

### 3.4. Association between the dietary OBS/lifestyle OBS and study outcomes

The associations of dietary and lifestyle OBS with study outcomes were also assessed (Table 5). Both dietary and lifestyle OBS performed flawlessly. For dietary OBS, the diagnosis of chronic bronchitis, cough, and wheezing, as well as the percent-predicted FVC, percent-predicted FEV1, and restrictive spirometry pattern, were statistically significant after adjustment.

**TABLE 5:** Associations between the dietary/lifestyle OBS and study outcomes.

	Unadjusted	*P*-value	Adjusted model 1	*P*-value	Adjusted model 2	*P*-value
**Dietary OBS**						
**Condition, OR (95% CI)**						
Asthma	0.996 (0.98 to 1.02)	0.699	0.99 (0.97 to 1.02)	0.420	0.99 (0.97 to 1.02)	0.420
Chronic bronchitis	0.96 (0.94 to 0.98)	<0.001	0.94 (0.91 to 0.97)	<0.001	0.95 (0.92 to 0.98)	0.001
**Symptom, OR (95% CI)**						
Cough	0.98 (0.96 to 1.01)	0.198	0.94 (0.92 to 0.97)	<0.001	0.95 (0.92 to 0.98)	<0.001
Phlegm production	0.98 (0.95 to 1.01)	0.185	0.96 (0.92 to 1.02)	0.161	0.97 (0.92 to 1.02)	0.189
Wheeze	0.96 (0.94 to 0.98)	<0.001	0.94 (0.92 to 0.96)	<0.001	0.95 (0.92 to 0.97)	<0.001
Exertional dyspnea	0.96 (0.93 to 1.00)	0.048	0.97 (0.92 to 1.02)	0.171	0.97 (0.92 to 1.02)	0.190
**Spirometry, MD (95% CI)**						
FEV1/FVC	−0.04 (−0.08 to 0.01)	0.123	0.04 (−0.002 to 0.09)	0.063	0.04 (−0.01 to 0.08)	0.104
Percent-predicted FVC	0.12 (0.06 to 0.18)	<0.001	0.13 (0.04 to 0.22)	0.004	0.11 (0.03 to 0.20)	0.012
Percent-predicted FEV1	0.13 (0.06 to 0.20)	<0.001	0.19 (0.08 to 0.29)	0.001	0.16 (0.06 to 0.26)	0.002
**Spirometry pattern, RRR (95% CI)**						
Obstructive	0.999 (0.98 to 1.02)	0.880	0.98 (0.96 to 1.01)	0.212	0.99 (0.96 to 1.01)	0.265
Restrictive	0.97 (0.94 to 0.99)	0.006	0.96 (0.92 to 0.99)	0.016	0.96 (0.93 to 0.997)	0.033
**Lifestyle OBS**						
**Condition, OR (95% CI)**						
Asthma	0.98 (0.92 to 1.04)	0.436	0.996 (0.93 to 1.06)	0.898	1.00 (0.94 to 1.07)	0.998
Chronic bronchitis	0.85 (0.75 to 0.96)	0.010	0.86 (0.76 to 0.99)	0.030	0.88 (0.77 to 1.01)	0.060
**Symptom, OR (95% CI)**						
Cough	0.87 (0.80 to 0.94)	0.001	0.92 (0.84 to 1.01)	0.075	0.94 (0.85 to 1.03)	0.147
Phlegm production	0.84 (0.75 to 0.95)	0.008	0.90 (0.77 to 1.04)	0.156	0.91 (0.78 to 1.05)	0.182
Wheeze	0.85 (0.80 to 0.90)	<0.001	0.88 (0.82 to 0.94)	<0.001	0.90 (0.84 to 0.96)	0.002
Exertional dyspnea	0.93 (0.82 to 1.04)	0.188	0.96 (0.85 to 1.09)	0.552	0.98 (0.87 to 1.12)	0.784
**Spirometry, MD (95% CI)**						
FEV1/FVC	−0.10 (−0.27 to 0.07)	0.251	0.15 (0.01 to 0.30)	0.043	0.14 (−0.01 to 0.28)	0.063
Percent-predicted FVC	0.47 (0.13 to 0.82)	0.009	0.45 (0.12 to 0.78)	0.008	0.41 (0.07 to 0.74)	0.018
Percent-predicted FEV1	0.66 (0.28 to 1.04)	<0.001	0.60 (0.23 to 0.97)	0.002	0.54 (0.18 to 0.90)	0.005
**Spirometry pattern, RRR (95% CI)**						
Obstructive	1.01 (0.95 to 1.08)	0.667	0.95 (0.88 to 1.02)	0.150	0.95 (0.89 to 1.02)	0.183
Restrictive	0.87 (0.79 to 0.96)	0.005	0.85 (0.77 to 0.94)	0.002	0.86 (0.78 to 0.96)	0.005

OR, odds ratio; MD, mean difference; RRR, relative risk ratio; CI, confidence intervals; OBS, oxidative balance score; FVC, forced vital capacity; FEV1, forced expiratory volume in one second.

The adjusted model 1 adjusted for age, sex, race/ethnicity, poverty-to-income ratio, and dietary energy. The adjusted model 2 in addition to adjusted for lifestyle OBS (or dietary OBS) based on adjusted model 1.

For lifestyle OBS, the results were similar to those of dietary OBS. The associations between lifestyle OBS and the diagnosis of asthma, cough, phlegm production, exertional dyspnea, obstructive spirometry pattern, as well as the diagnosis of chronic bronchitis and FEV1/FVC were not statistically significant, but other outcomes were statistically significant. Notably, a significantly higher percent-predicted FVC and FEV1 was found in those with higher lifestyle OBS and dietary OBS (Table 5).

Furthermore, interactions between dietary OBS and lifestyle were also assessed (e.g., *P* for interaction = 0.017 for dietary OBS * lifestyle OBS on the diagnosis of chronic bronchitis) (Supplementary Table 1). Interestingly, both diet OBS and lifestyle OBS deserve attention.

### 3.5. Additional analysis

Table 6 shows the associations of OBS with study outcomes in male and female subjects. For instance, a one-unit increase in OBS was associated with a 0.25% (95% CI: 0.14, 0.36) increase in percent-predicted FEV1 among male subjects and 0.19% (95% CI: 0.03, 0.35) increase in percent-predicted FEV1 among female subjects. There was a significant association between OBS and the diagnosis of wheezing both in male and female subjects (Table 6). However, there were gender differences in the association between OBS and the diagnosis of chronic bronchitis, cough, exertional dyspnea, percent-predicted FVC and restrictive spirometry pattern. Additionally, *P* for interaction = 0.005 for OBS * sex on the restrictive spirometry pattern.

**TABLE 6:** Effect modification of oxidative balance score on study outcomes by sex.

	Male		Female		*P* for interaction
	Adjusted model	*P*-value	Adjusted model	*P*-value	
**Condition, OR (95% CI)**					
Asthma	0.997 (0.96 to 1.03)	0.860	0.99 (0.97 to 1.01)	0.354	0.302
Chronic bronchitis	0.95 (0.89 to 1.01)	0.106	0.94 (0.90 to 0.97)	<0.001	0.442
**Symptom, OR (95% CI)**					
Cough	0.94 (0.89 to 0.99)	0.015	0.95 (0.90 to 1.01)	0.088	0.613
Phlegm production	0.95 (0.90 to 1.003)	0.064	0.97 (0.89 to 1.06)	0.507	0.663
Wheeze	0.93 (0.90 to 0.96)	<0.001	0.95 (0.93 to 0.97)	<0.001	0.844
Exertional dyspnea	1.00 (0.93 to 1.08)	0.998	0.93 (0.88 to 0.99)	0.024	0.471
**Spirometry, MD (95% CI)**					
FEV1/FVC	0.04 (−0.003 to 0.09)	0.068	0.06 (−0.01 to 0.13)	0.098	0.719
Percent-predicted FVC	0.19 (0.10 to 0.29)	<0.001	0.12 (−0.01 to 0.26)	0.077	0.182
Percent-predicted FEV1	0.25 (0.14 to 0.36)	<0.001	0.19 (0.03 to 0.35)	0.023	0.225
**Spirometry pattern, RRR (95% CI)**					
Obstructive	0.99 (0.96 to 1.01)	0.286	0.97 (0.94 to 1.01)	0.189	0.148
Restrictive	0.92 (0.89 to 0.96)	<0.001	0.98 (0.92 to 1.04)	0.458	0.003

OR, odds ratio; MD, mean difference; RRR, relative risk ratio; CI, confidence intervals; FVC, forced vital capacity; FEV1, forced expiratory volume in one second.

The adjusted models were adjusted by age, sex, race/ethnicity, poverty-to-income ratio, and dietary energy.

In sensitivity analyses, when interview weight was used instead of dietary weight, the results remained unchanged in the following cases: (1) re-examining the associations of OBS with study outcomes (Supplementary Table 2); (2) re-examining the associations of quartiles of OBS on study outcomes (Supplementary Table 3); and (3) re-examining the associations between the dietary/lifestyle OBS and study outcomes (Supplementary Table 4).

## 4. Discussion

In order to elucidate the relationship between OBS and lung health, a cross-sectional analysis of 5,214 individuals in the NHANES cohort was performed. The total OBS was negatively associated with the diagnosis of chronic bronchitis, the symptom of wheezing, and restrictive spirometry pattern and was also positively associated with percent-predicted FEV1 and FVC. There was sexual dimorphism among these associations. Furthermore, both an antioxidant diet and an antioxidant lifestyle were critical in lung health. Overall, the higher the OBS score, the better the pulmonary outcomes. Our results highlight the importance of adherence to an antioxidant diet and lifestyle, particularly for lung function.

The association between the OBS and lung health was stable. Although there was no direct evidence, several studies have been conducted to explore the relationships between diet and respiratory diseases. Previous studies have reported that dietary vitamin C could prevent COPD (29), and dietary fiber could reduce the prevalence of COPD (14). In patients with COPD, especially smokers, reduced dietary intake of vitamin C and β-carotene levels were linked with reduced FEV1 and FVC as well as bronchiectasis symptoms (30, 31). In addition, dietary vitamin A might relieve airway obstruction in smokers (32). Dietary fiber was independently related to improved lung function (14).

Studies on serum antioxidant nutrients could also serve as a basis for support. For example, lower plasma carotene levels (33) and lower plasma ascorbic acid levels (34, 35) were both associated with increased odds of asthma diagnosis. Meanwhile, reduced ascorbic acid levels were also related to increased respiratory symptoms (33). Decreased ascorbic acid levels were associated with diminished lung function (33). Moreover, each of the dietary and serum antioxidant nutrients was significantly associated with FEV1 (36). Furthermore, there were several experimental studies that provided a convincing argument that vitamin C could prevent smoke-induced oxidative stress and provide pulmonary restoration in mice (37).

The association between lifestyle and lung health has also been demonstrated. Several studies have revealed higher levels of airway oxidative stress biomarkers as BMI increased (38, 39). Airway oxidative stress associated with obesity and occurring after a high-fat diet has been well documented (40, 41). But in patients with asthma, increased BMI was related to reduced airway eosinophils and lower exhaled nitric oxide levels, which represented no more airway inflammation in patients (42, 43). However, the fact that exercise interventions appeared to affect the redox capacity of patients with COPD has suggested a lack of high-quality evidence (44).

Other chronic disease studies suggested that a combination of factors may be more strongly correlated with disease risk than any nutrient considered alone (45, 46). Incorporating pro-oxidant and antioxidant exposures into composite measures of oxidative balance may be more strongly correlated with health outcomes than any factor considered alone (47, 48). Therefore, in the present study, we used the OBS based on the total intake of various pro-oxidants and antioxidants on the overall measure of oxidative stress-related exposures.

Our results showed sexual dimorphism in the effect of OBS on the respiratory system. Some factors could contribute to this condition. First, healthy women have smaller central airways than men over a range of ages (49), and thus lung function may be influenced by gender-related biological differences (50). Second, men smoked more intensely than women and were more likely to benefit from dietary antioxidants due to increased levels of oxidative stress from smoking and the oxidative burden that remains even after quitting compared with never smoking (51). Third, women are usually exposed to different respiratory risk factors than men. Our data showed that the impact of OBS on respiratory disease varied by gender, which strengthened the relevance of OBS when assessing lung health.

The association between OBS and the diagnosis of cough/exertional dyspnea, as well as FEV1/FVC, differed between the unadjusted and adjusted models. Possible reasons for this were as follows: 1) limitations in the number of people involved. There were only 245 patients with coughing most days in over 3 months and 299 patients with exertional dyspnea. The results might be biased and need to be further explored and confirmed in the future with a large sample. 2) OBS was not an independent influence on these outcomes and could be affected by other confounding factors. Such interactions need to be further explored because they are so extensive.

This study has several strengths. First, our study focused on OBS rather than a single component to comprehensively capture the complex relationships among various factors and to provide a more comprehensive investigation of relevant outcomes. Second, NAHANES data have been collected through thorough quality control and quality assurance procedures so that the data could represent a sample of the deinstitutionalized civilian population. Thus, the results could be generalized to all deinstitutionalized civilians in the United States. Third, the outcomes included in our study were exhaustive, and the outcomes included respiratory symptoms, chronic lung disease, and lung function. Therefore, in terms of respiratory disease prevention, the findings have more public health implications.

The study also had several limitations. First, due to the cross-sectional nature of the data, it is hard to establish a causal relationship between OBS and respiratory diseases. Therefore, more prospectively designed studies are necessary to validate the effectiveness of OBS. Second, due to database limitations, the number of patients with asthma, obstructive spirometry pattern, or restrictive spirometry pattern was limited; and factors included in the OBS were incomplete; for example, many components, including flavonoids and some ambiguous dietary or lifestyle exposures, could not be collected. However, the relationship between lung health and OBS was robust enough, according to the results of this study. Third, under the assumption that all pro-oxidants and antioxidants were linearly related to oxidative stress, the threshold effect of antioxidants (52, 53) was neglected in this study. However, certain antioxidants have been demonstrated to potentially exhibit pro-oxidant activity at high doses or under certain conditions. Fourth, the long-term dynamic variation of OBS components, including lifestyle components, cannot be accurately measured and collected in the present study. Fifth, the covariates included in this study were incomplete, and some unmeasured confounding factors were not mentioned. Variables for medication use were not considered due to data limitations.

## 5. Conclusion

In conclusion, a higher OBS, indicating more significant antioxidant exposures, was strongly negatively associated with the diagnosis of chronic bronchitis, the symptom of wheezing, and restrictive spirometry pattern and was positively associated with percent-predicted FVC and FEV1. Both antioxidant diet and lifestyle improvements were effective when it came to preventing lung health. But there was sexual dimorphism among these associations. The results highlight the importance of adherence to an antioxidant diet and lifestyle and that it contributes to lung health. In the future, further understanding of the temporality of the associations between OBS and lung health, especially in the context of established respiratory disease, and investigation of the precise mechanisms behind these associations should be pursued.

## Data availability statement

This study used data from the National Health and Nutrition Examination Survey (NHANES) (https://www.cdc.gov/nchs/nhanes/index.htm).

## Ethics statement

The National Health and Nutrition Examination Survey involving human participants was reviewed and approved by the National Center for Health Statistics Ethics Review Board. The patients/participants provided their written informed consent. This study involved secondary data analysis of the National Health and Nutrition Examination Survey, and this study we conducted was exempt from institutional review for this reason.

## Author contributions

CC: design of this study and manuscript revision. ZX: data collection and analysis and manuscript drafting. YX: data analysis and manuscript revision. HW: data curation and manuscript revision. All authors contributed to the article and approved the submitted version.
