# Supplementary material for: Association of oxidative balance score and lung health from the National Health and Nutrition Examination Survey 2007–2012
Source: Front Nutr. 2023 Jan 9;9:961950. doi: 10.3389/fnut.2022.961950 (PMC9869685; doi:10.3389/fnut.2022.961950)
Supplement: Supplementary file 1 [file Table_1.DOCX]

Supplementary Material

Association of Oxidative Balance Score and Lung Health from the National Health and Nutrition Examination Survey 2007-2012

Zhixiao Xu, Yincong Xue, Hezhi Wen, Chengshui Chen*

*** Correspondence:** Corresponding Author: Chengshui Chen; E-mail: chenchengshui@wmu.edu.cn

Table S1. The interaction P values between oxidative balance score and lung health.

|  | Dietary OBS * Lifestyle OBS | | |
| --- | --- | --- | --- |
|  | All | Male | Female |
| **Condition** | | |  |
| Asthma | 0.863 | 0.843 | 0.997 |
| Chronic bronchitis | **0.017** | 0.177 | **0.031** |
| **Symptom** | | |  |
| Cough | 0.566 | 0.975 | 0.382 |
| Phlegm production | 0.725 | 0.621 | 0.244 |
| Wheeze | 0.211 | 0.272 | **0.005** |
| Exertional dyspnea | 0.834 | **0.020** | 0.075 |
| **Spirometry** | | |  |
| FEV1/FVC | 0.950 | 0.137 | 0.266 |
| FVCpp | 0.905 | 0.275 | 0.313 |
| FEV1pp | 0.889 | 0.655 | 0.892 |
| **Spirometry pattern** | | |  |
| Obstructive | 0.977 | 0.787 | 0.898 |
| Restrictive | 0.631 | 0.636 | 0.463 |

FEV1pp: percent-predicted FEV1; FVCpp: percent-predicted FVC; OBS: oxidative balance score.

The adjusted models were adjusted by age, sex, race/ethnicity, poverty-income ratio and dietary energy.

Table S2. Sensitivity analysis by replacing the dietary weight with the interview weight: association of oxidative balance score with study outcomes.

|  |  | Unadjusted | P-value |  | Adjusted | P-value |
| --- | --- | --- | --- | --- | --- | --- |
| **Condition, OR (95% CI)** | | |  |  |  |  |
| Asthma |  | 0.99 (0.97 to 1.01) | 0.249 |  | 0.99 (0.97 to 1.01) | 0.260 |
| Chronic bronchitis | | 0.96 (0.94 to 0.98) | <0.001 |  | 0.95 (0.91 to 0.98) | 0.004 |
| **Symptom, OR (95% CI)** | | |  |  |  |  |
| Cough |  | 0.97 (0.95 to 0.997) | 0.028 |  | 0.95 (0.92 to 0.98) | <0.001 |
| Phlegm production | | 0.97 (0.95 to 0.996) | 0.025 |  | 0.96 (0.92 to 1.003) | 0.07 |
| Wheeze |  | 0.96 (0.95 to 0.98) | <0.001 |  | 0.95 (0.93 to 0.97) | <0.001 |
| Exertional dyspnea | | 0.95 (0.93 to 0.98) | 0.001 |  | 0.95 (0.92 to 0.99) | 0.028 |
| **Spirometry, MD (95% CI)** | | |  |  |  |  |
| FEV1/FVC |  | -0.04 (-0.08 to -0.002) | 0.042 |  | 0.05 (0.02 to 0.09) | 0.007 |
| FVCpp |  | 0.13 (0.08 to 0.19) | <0.001 |  | 0.16 (0.08 to 0.23) | <0.001 |
| FEV1pp |  | 0.15 (0.08 to 0.21) | <0.001 |  | 0.22 (0.12 to 0.31) | <0.001 |
| **Spirometry pattern, RRR (95% CI)** | | |  |  |  |  |
| Obstructive | | 1.001 (0.99 to 1.02) | 0.900 |  | 0.98 (0.96 to 0.998) | 0.035 |
| Restrictive |  | 0.97 (0.95 to 0.99) | 0.006 |  | 0.95 (0.92 to 0.98) | 0.003 |

OR: odds ratio; MD: mean difference; RRR: relative risk ratio; CI: confidence intervals; FEV1pp: percent-predicted FEV1; FVCpp: percent-predicted FVC; OBS: oxidative balance score.

The adjusted models were adjusted by age, sex, race/ethnicity, poverty-income ratio and dietary energy.

Table S3. Sensitivity analysis by replacing the dietary weight with the interview weight: effect modification of quartiles of OBS on study outcomes.

|  | Unadjusted | P-value |  | Adjusted | P-value |
| --- | --- | --- | --- | --- | --- |
| **Condition, OR (95% CI)** | |  |  |  |  |
| Asthma |  |  |  |  |  |
| OBS per SD | 0.93 (0.82 to 1.05) | 0.249 |  | 0.92 (0.79 to 1.07) | 0.260 |
| Q1 | Ref | Ref |  | Ref | Ref |
| Q2 | 0.85 (0.64 to 1.11) | 0.226 |  | 0.85 (0.63 to 1.16) | 0.303 |
| Q3 | 0.92 (0.68 to 1.25) | 0.593 |  | 0.93 (0.67 to 1.29) | 0.646 |
| Q4 | 0.79 (0.57 to 1.09) | 0.148 |  | 0.76 (0.51 to 1.13) | 0.166 |
| P for trend | | 0.198 |  |  | 0.204 |
| Chronic bronchitis | |  |  |  |  |
| OBS per SD | 0.73 (0.62 to 0.87) | <0.001 |  | 0.68 (0.53 to 0.88) | 0.004 |
| Q1 | Ref | Ref |  | Ref | Ref |
| Q2 | 0.47 (0.28 to 0.78) | 0.004 |  | 0.44 (0.26 to 0.77) | 0.005 |
| Q3 | 0.58 (0.38 to 0.91) | 0.019 |  | 0.55 (0.33 to 0.93) | 0.025 |
| Q4 | 0.41 (0.23 to 0.72) | 0.002 |  | 0.33 (0.15 to 0.75) | 0.009 |
| P for trend | | 0.001 |  |  | 0.008 |
| **Symptom, OR (95% CI)** | |  |  |  |  |
| Cough |  |  |  |  |  |
| OBS per SD | 0.82 (0.69 to 0.98) | 0.028 |  | 0.69 (0.56 to 0.85) | <0.001 |
| Q1 | Ref | Ref |  | Ref | Ref |
| Q2 | 1.08 (0.69 to 1.68) | 0.727 |  | 0.96 (0.63 to 1.46) | 0.833 |
| Q3 | 0.79 (0.52 to 1.22) | 0.281 |  | 0.63 (0.41 to 0.96) | 0.034 |
| Q4 | 0.66 (0.38 to 1.15) | 0.138 |  | 0.45 (0.25 to 0.82) | 0.011 |
| P for trend | | 0.057 |  |  | 0.003 |
| Phlegm production | |  |  |  |  |
| OBS per SD | 0.81 (0.68 to 0.973) | 0.025 |  | 0.75 (0.54 to 1.03) | 0.07 |
| Q1 | Ref | Ref |  | Ref | Ref |
| Q2 | 1.07 (0.65 to 1.77) | 0.776 |  | 1.09 (0.64 to 1.85) | 0.745 |
| Q3 | 0.70 (0.41 to 1.21) | 0.194 |  | 0.67 (0.35 to 1.31) | 0.239 |
| Q4 | 0.70 (0.42 to 1.17) | 0.171 |  | 0.63 (0.28 to 1.42) | 0.259 |
| P for trend | | 0.070 |  |  | 0.154 |
| Wheeze |  |  |  |  |  |
| OBS per SD | 0.75 (0.67 to 0.85) | <0.001 |  | 0.68 (0.58 to 0.79) | <0.001 |
| Q1 | Ref | Ref |  | Ref | Ref |
| Q2 | 0.74 (0.58 to 0.93) | 0.011 |  | 0.68 (0.52 to 0.89) | 0.006 |
| Q3 | 0.59 (0.46 to 0.77) | <0.001 |  | 0.51 (0.38 to 0.68) | <0.001 |
| Q4 | 0.47 (0.34 to 0.67) | <0.001 |  | 0.36 (0.23 to 0.57) | <0.001 |
| P for trend | | <0.001 |  |  | <0.001 |
| Exertional dyspnea | |  |  |  |  |
| OBS per SD | 0.71 (0.58 to 0.87) | 0.001 |  | 0.72 (0.53 to 0.96) | 0.028 |
| Q1 | Ref | Ref |  | Ref | Ref |
| Q2 | 0.50 (0.31 to 0.81) | 0.006 |  | 0.55 (0.32 to 0.95) | 0.032 |
| Q3 | 0.53 (0.33 to 0.84) | 0.008 |  | 0.53 (0.30 to 0.94) | 0.030 |
| Q4 | 0.40 (0.22 to 0.71) | 0.003 |  | 0.42 (0.19 to 0.92) | 0.032 |
| P for trend | | 0.001 |  |  | 0.023 |
| **Spirometry, MD (95% CI)** | |  |  |  |  |
| FEV1/FVC |  |  |  |  |  |
| OBS per SD | -0.3 (-0.59 to -0.01) | 0.042 |  | 0.39 (0.12 to 0.66) | 0.007 |
| Q1 | Ref | Ref |  | Ref | Ref |
| Q2 | -0.20 (-0.88 to 0.49) | 0.567 |  | 0.68 (0.04 to 1.33) | 0.038 |
| Q3 | -0.58 (-1.30 to 0.13) | 0.108 |  | 0.72 (0.09 to 1.34) | 0.025 |
| Q4 | -0.66 (-1.46 to 0.14) | 0.102 |  | 1.06 (0.33 to 1.80) | 0.006 |
| P for trend | | 0.076 |  |  | 0.012 |
| FVCpp |  |  |  |  |  |
| OBS per SD | 0.95 (0.56 to 1.34) | <0.001 |  | 1.11 (0.57 to 1.65) | <0.001 |
| Q1 | Ref | Ref |  | Ref | Ref |
| Q2 | 0.33 (-0.73 to 1.39) | 0.534 |  | 0.61 (-0.58 to 1.79) | 0.305 |
| Q3 | 1.56 (0.56 to 2.56) | 0.003 |  | 1.84 (0.63 to 3.04) | 0.004 |
| Q4 | 2.30 (1.24 to 3.37) | <0.001 |  | 2.61 (1.05 to 4.17) | 0.002 |
| P for trend | | <0.001 |  |  | <0.001 |
| FEV1pp |  |  |  |  |  |
| OBS per SD | 1.05 (0.58 to 1.52) | <0.001 |  | 1.56 (0.89 to 2.24) | <0.001 |
| Q1 | Ref | Ref |  | Ref | Ref |
| Q2 | 0.86 (-0.36 to 2.07) | 0.162 |  | 1.46 (0.20 to 2.72) | 0.025 |
| Q3 | 1.85 (0.62 to 3.09) | 0.004 |  | 2.68 (1.25 to 4.12) | <0.001 |
| Q4 | 2.68 (1.37 to 3.99) | <0.001 |  | 3.88 (2.03 to 5.74) | <0.001 |
| P for trend | | <0.001 |  |  | <0.001 |
| **Spirometry pattern, RRR (95% CI)** | |  |  |  |  |
| Obstructive | |  |  |  |  |
| OBS per SD | 1.01 (0.90 to 1.12) | 0.900 |  | 0.85 (0.72 to 0.99) | 0.035 |
| Q1 | Ref | Ref |  | Ref | Ref |
| Q2 | 0.95 (0.73 to 1.25) | 0.728 |  | 0.77 (0.57 to 1.03) | 0.080 |
| Q3 | 1.07 (0.80 to 1.42) | 0.669 |  | 0.78 (0.56 to 1.09) | 0.151 |
| Q4 | 1.02 (0.75 to 1.38) | 0.905 |  | 0.69 (0.47 to 1.01) | 0.055 |
| P for trend | | 0.747 |  |  | 0.111 |
| Restrictive |  |  |  |  |  |
| OBS per SD | 0.80 (0.68 to 0.94) | 0.006 |  | 0.70 (0.56 to 0.88) | 0.003 |
| Q1 | Ref | Ref |  | Ref | Ref |
| Q2 | 0.79 (0.50 to 1.27) | 0.336 |  | 0.67 (0.41 to 1.08) | 0.103 |
| Q3 | 0.63 (0.42 to 0.94) | 0.025 |  | 0.49 (0.30 to 0.78) | 0.003 |
| Q4 | 0.49 (0.3 to 0.807) | 0.005 |  | 0.34 (0.18 to 0.64) | <0.001 |
| P for trend | | 0.001 |  |  | <0.001 |

OR: odds ratio; MD: mean difference; RRR: relative risk ratio; CI: confidence intervals; FEV1pp: percent-predicted FEV1; FVCpp: percent-predicted FVC; OBS: oxidative balance score.

The adjusted models were adjusted by age, sex, race/ethnicity, poverty-income ratio and dietary energy.

Table S4. Sensitivity analysis by replacing the dietary weight with the interview weight: associations between the dietary/lifestyle OBS and study outcomes.

|  |  | Unadjusted | P-value |  | Adjusted Model 1 | P-value |  | Adjusted Model 2 | P-value |
| --- | --- | --- | --- | --- | --- | --- | --- | --- | --- |
| **Dietary OBS** | |  |  |  |  |  |  |  |  |
| **Condition, OR (95% CI)** | | |  |  |  |  |  |  |  |
| Asthma |  | 0.99 (0.97 to 1.01) | 0.401 |  | 0.99 (0.97 to 1.01) | 0.38 |  | 0.99 (0.97 to 1.02) | 0.457 |
| Chronic bronchitis | | 0.96 (0.94 to 0.98) | 0.001 |  | 0.95 (0.92 to 0.99) | 0.008 |  | 0.96 (0.92 to 0.99) | 0.015 |
| **Symptom, OR (95% CI)** | | |  |  |  |  |  |  |  |
| Cough |  | 0.98 (0.95 to 1.01) | 0.109 |  | 0.95 (0.92 to 0.98) | 0.002 |  | 0.95 (0.92 to 0.98) | 0.004 |
| Phlegm production | | 0.98 (0.95 to 1.01) | 0.116 |  | 0.96 (0.92 to 1.01) | 0.111 |  | 0.97 (0.93 to 1.01) | 0.142 |
| Wheeze |  | 0.97 (0.95 to 0.98) | <0.001 |  | 0.95 (0.93 to 0.97) | <0.001 |  | 0.95 (0.93 to 0.98) | <0.001 |
| Exertional dyspnea | | 0.95 (0.92 to 0.98) | <0.001 |  | 0.95 (0.91 to 0.99) | 0.015 |  | 0.95 (0.91 to 0.99) | 0.013 |
| **Spirometry, MD (95% CI)** | | |  |  |  |  |  |  |  |
| FEV1/FVC |  | -0.04 (-0.08 to 0.002) | 0.062 |  | 0.05 (0.01 to 0.09) | 0.008 |  | 0.05 (0.01 to 0.09) | 0.011 |
| FVCpp |  | 0.12 (0.06 to 0.18) | <0.001 |  | 0.14 (0.06 to 0.22) | 0.001 |  | 0.12 (0.04 to 0.2) | 0.006 |
| FEV1pp |  | 0.13 (0.06 to 0.19) | <0.001 |  | 0.20 (0.11 to 0.29) | <0.001 |  | 0.18 (0.09 to 0.27) | <0.001 |
| **Spirometry pattern, RRR (95% CI)** | | |  |  |  |  |  |  |  |
| Obstructive | | 0.999 (0.98 to 1.01) | 0.919 |  | 0.98 (0.96 to 0.999) | 0.036 |  | 0.98 (0.96 to 0.999) | 0.042 |
| Restrictive |  | 0.97 (0.95 to 0.998) | 0.036 |  | 0.96 (0.93 to 0.99) | 0.022 |  | 0.97 (0.94 to 1) | 0.052 |
|  |  |  |  |  |  |  |  |  |  |
| **Lifestyle OBS** | |  |  |  |  |  |  |  |  |
| **Condition, OR (95% CI)** | | |  |  |  |  |  |  |  |
| Asthma |  | 0.95 (0.90 to 0.998) | 0.041 |  | 0.96 (0.91 to 1.02) | 0.196 |  | 0.97 (0.92 to 1.02) | 0.249 |
| Chronic bronchitis | | 0.86 (0.76 to 0.97) | 0.012 |  | 0.88 (0.78 to 1.001) | 0.051 |  | 0.90 (0.79 to 1.02) | 0.09 |
| **Symptom, OR (95% CI)** | | |  |  |  |  |  |  |  |
| Cough |  | 0.86 (0.81 to 0.93) | <0.001 |  | 0.92 (0.85 to 0.98) | 0.018 |  | 0.93 (0.87 to 0.997) | 0.041 |
| Phlegm production | | 0.84 (0.76 to 0.94) | 0.004 |  | 0.89 (0.78 to 1.02) | 0.102 |  | 0.90 (0.79 to 1.03) | 0.123 |
| Wheeze |  | 0.86 (0.82 to 0.90) | <0.001 |  | 0.89 (0.84 to 0.94) | <0.001 |  | 0.91 (0.86 to 0.96) | <0.001 |
| Exertional dyspnea | | 0.94 (0.84 to 1.05) | 0.293 |  | 0.98 (0.86 to 1.11) | 0.769 |  | 1.01 (0.90 to 1.15) | 0.817 |
| **Spirometry, MD (95% CI)** | | |  |  |  |  |  |  |  |
| FEV1/FVC |  | -0.15 (-0.30 to 0.002) | 0.052 |  | 0.11 (-0.02 to 0.25) | 0.100 |  | 0.09 (-0.04 to 0.23) | 0.168 |
| FVCpp |  | 0.47 (0.19 to 0.74) | 0.001 |  | 0.45 (0.19 to 0.71) | 0.001 |  | 0.40 (0.14 to 0.66) | 0.004 |
| FEV1pp |  | 0.61 (0.30 to 0.92) | <0.001 |  | 0.55 (0.25 to 0.85) | <0.001 |  | 0.48 (0.19 to 0.77) | 0.002 |
| **Spirometry pattern, RRR (95% CI)** | | |  |  |  |  |  |  |  |
| Obstructive | | 1.03 (0.97 to 1.09) | 0.327 |  | 0.96 (0.90 to 1.03) | 0.231 |  | 0.97 (0.91 to 1.03) | 0.338 |
| Restrictive |  | 0.86 (0.79 to 0.93) | <0.001 |  | 0.84 (0.78 to 0.92) | <0.001 |  | 0.85 (0.79 to 0.93) | <0.001 |

OR: odds ratio; MD: mean difference; RRR: relative risk ratio; CI: confidence intervals; FEV1pp: percent-predicted FEV1; FVCpp: percent-predicted FVC; OBS: oxidative balance score.

The adjusted model 1 adjusted for age, sex, race/ethnicity, poverty-income ratio and dietary energy. The adjusted model 2 additionally adjusted for lifestyle OBS (or dietary OBS) based on adjusted model 1.
